# Time-Dependent Flow of Water-Based CoFe_2_O_4_-Mn-ZnFe_2_O_4_ Nanoparticles over a Shrinking Sheet with Mass Transfer Effect in Porous Media

**DOI:** 10.3390/nano12224102

**Published:** 2022-11-21

**Authors:** Iskandar Waini, Umair Khan, Aurang Zaib, Anuar Ishak, Ioan Pop, Nevzat Akkurt

**Affiliations:** 1 Fakulti Teknologi Kejuruteraan Mekanikal dan Pembuatan, Universiti Teknikal Malaysia Melaka, Hang Tuah Jaya, Durian Tunggal, Melaka 76100, Malaysia; 2 Department of Mathematical Sciences, Faculty of Science and Technology, Universiti Kebangsaan Malaysia, Bangi 43600, Malaysia; 3 Department of Mathematics and Social Sciences, Sukkur IBA University, Sukkur 65200, Pakistan; 4 Department of Mathematical Sciences, Federal Urdu University of Arts, Science & Technology, Gulshan-e-Iqbal, Karachi 75300, Pakistan; 5 Department of Mathematics, Babes-Bolyai University, 400084 Cluj-Napoca, Romania; 6 Academy of Romanian Scientists, 3 Ilfov Street, 050044 Bucharest, Romania; 7 Rare Earth Elements Application and Research Center, Munzur University, 62000 Tunceli, Turkey

**Keywords:** hybrid nanofluid, ferrite nanoparticle, porous medium, shrinking sheet, unsteady flow

## Abstract

The use of hybrid nanoparticles to increase heat transfer is a favorable area of research, and therefore, numerous scientists, researchers, and scholars have expressed their appreciation for and interest in this field. Determining the dynamic role of nanofluids in the cooling of microscopic electronic gadgets, such as microchips and related devices, is also one of the fundamental tasks. With such interesting and useful applications of hybrid nanofluids in mind, the main objective is to deal with the analysis of the unsteady flow towards a shrinking sheet in a water-based hybrid ferrite nanoparticle in porous media, with heat sink/source effects. Moreover, the impact of these parameters on heat and mass transfers is also reported. Numerical results are obtained using MATLAB software. Non-unique solutions are determined for a certain shrinking strength, in addition to the unsteadiness parameter. The mass transfer and friction factor increase for the first solution due to the hybrid nanoparticles, but the heat transfer rate shows the opposite effect.

## 1. Introduction

Heat transfer through heat exchangers in multi-phase flow applications is among the most important processes in industry. Despite the built-in and well-developed models that have been applied since the 1970s, much attention has been focused on the experimental works, due to the needs and strong desires from industry. In recent years, considerable efforts have been expended toward improving the phenomenon of heat transport, one of which is to improve the thermal conductivity. Earlier researchers, such as Choi and Eastman [1], conducted a study of nanofluids and their capabilities regarding heat transmission. Saidur et al. [2] presented the new properties of fluids that enable the achievement of incredible advances in a variety of applications in industry, including hybrid motors, domestic refrigerators, microelectronics, and coolers. Motsumi and Makinde [3] investigated the radiative flow over a moving porous plate using nanofluid. Sheikholeslami [4] investigated the heat transmission of a nanofluid from a cylinder using a steady suction and explained the escalating function of the Nusselt number in conjunction with the percentage of nanoparticles. The heat transmission in a nanofluid passed over a porous shrinkable/stretchable sheet with the magnetic field was analyzed by Naramgari and Sulochana [5], which indicated that the drag force and heat transport phenomenon is bounded by the magnetic field. Other research regarding nanofluids can be found in recent articles [6,7,8,9,10].

Recently, researchers have paid particular attention to hybrid nanofluids, and many reports have suggested that hybrid nanofluids may improve the heat transfer rate and reduce production costs compared to the use of ordinary nanofluids, benefits that can lead to efficient outputs [11]. Hybrid nanofluids are formed by mixing two or more types of nanometer-sized particles in the base fluids. The preparation of hybrid nanofluids, as well as the discussion on their thermal properties, was conducted by Sundar et al. [12]. Devi and Devi [13] examined the heat transfer enrichment induced by stretchable sheets of dispersed water-based alumina and copper hybrid nanoparticles. Yousefi et al. [14] explored the copper-titania nanoparticles flowing towards a stagnation region of a wavy cylinder. Waini et al. [15] investigated the time-varying flow and heat transfer over shrinkable/stretchable surfaces in hybrid nanofluids. They found non-unique solutions for a particular range of the moving parameter. Sreedevi et al. [16] examined the radiative consequences of time-dependent flow on a stretched sheet induced by a hybrid nanofluid with slip effects. Recently, some researchers [17,18,19,20,21] debated the significance of hybrid nanofluids from different aspects. Valuable references on hybrid nanofluids can be also found in the book by Merkin et al. [22].

The various potential and actual thermal engineering applications of porous media flow in areas such as packed bed reactors, thermal insulation systems, heat storage beds, geothermal engineering, petroleum recovery, groundwater pollution, and ceramic processing frequently motivate interest in such studies. Several investigations have been conducted on the flow with the problems of mass and heat transfer in homogeneous saturated porous media. Abel et al. [23] examined the viscoelastic fluid flow, with heat transport rate, past a non-isothermal stretchable surface subjected to the impacts of the porous medium. Ahmad and Pop [24] studied the mixed convective flow from a smooth plate in a porous medium. Pal and Mondal [25] investigated the Dufour and Soret diffusion effects on a radiative flow induced by a stretchable surface with a chemical reaction in porous media. They scrutinized that the heat uplifts due to the Dufour, as well as the radiation, effect. The impact of suction on a micropolar fluid flow through a stretchable sheet was studied by Rosali et al. [26], and they reported double solutions. Vyas and Srivastava [27] examined the flow over a shrinkable sheet in a porous media with radiative heat transfer and reported double solutions for certain values of suction. The forced convection flow in Darcy–Brinkman porous media was inspected by Pantokratoras [28]. Kumar and Sood [29] examined the magnetic effect on the Darcy—Brinkman—Forchheimer flow over a shrinkable sheet in a porous medium with mixed convection. Hussanan et al. [30] reported an analytic solution for the time-varying magnetohydrodynamic flow of a Casson liquid via a vertical oscillating infinite plate-saturated porous media. Hassan et al. [31] scrutinized the flow of convective and heat transport rate comprising porous media, with water-based nanofluid, past a wavy surface by considering copper oxide nanoparticles. The entropy impact on the buoyancy or mixed convective nanofluid flow induced by micropolar fluid towards a vertical plate comprising a non-Darcy medium was inspected by Zaib et al. [32]. Recently, Khan et al. [33] considered the buoyancy effects on micropolar fluid comprising porous media through a vertical plate containing hybrid nanoparticles and reported double solutions.

The study of mass and heat transmission using hybrid nanofluid has been disclosed to be reasonably important in different engineering processes, such as heating development, biomedical processes, polymer extrusion, and many more. Thus, the present work intends to study the unsteady flow over a stretchable/shrinkable sheet in a saturated porous medium containing ferrite hybrid nanoparticles. Tiwari and Das [34] models are utilized to propose the hybrid nanofluid model. By using a numerical approach, the computed dual solutions of the transformed equations are provided. The behavior of the flow, combined with the characteristics of mass and heat transfers, are inspected for plentiful parameters, which are depicted in graphical and tabular forms.

## 2. Description and Framework of the Mathematical Model

**Figure 1:**
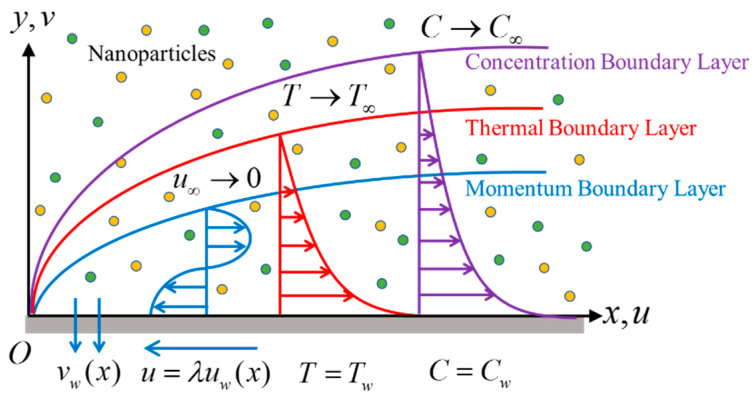
Physical flow configuration.

Consider the time-dependent flow past a shrinking continuously sheet in the presence of a hybrid nanofluid, as shown in Figure 1, where (x,y) are the Cartesian coordinates, with x—axis measured along the surface of the sheet, and the y—axis normal to it, the flow being y≥0. The stretching velocity is assumed as $u_{w}(x,t)=ax/\left(1-\alpha t\right)$, where t is the time, while a and α are constants, while the velocity of the mass flux is $v_{w}(t)$. Two types of nanoparticles are studied, namely cobalt ferrite (CoFe_2_O_4_) and manganese-zinc ferrite (Mn-ZnFe_2_O_4_), which are dispersed in the water-based fluid. For the hybrid nanofluid, it is assumed that the size of the nanoparticles is uniform, and the effect of the agglomeration of the nanoparticles is neglected because the nanofluids are synthesized as a stable mixture of the base fluid and the nanoparticles. The governing equations of the conservation of mass, momentum, energy, and concentration by using the usual boundary layer approximations are written as [15,35]:

(1) $$\frac{\partial u\;}{\partial x}+\frac{\partial v}{\partial y}=0,$$

(2) $$\rho_{hnf}\left(\frac{\partial u}{\partial t}+u\frac{\partial u}{\partial x}+v\frac{\partial u}{\partial y}\right)=\mu_{hnf}\;\left(\frac{\partial^{2}u}{\partial y^{2}}-\frac{u}{K_{0}}\right),$$

(3) $${\left(\rho C_{p}\right)}_{hnf}\left(\frac{\partial T}{\partial t}+u\frac{\partial T}{\partial x}+v\frac{\partial T}{\partial y}\right)=k_{hnf}\frac{\partial^{2}T}{\partial y^{2}}+Q_{0}\left(T-T_{\infty }\right),$$

(4) $$\frac{\partial C}{\partial t}+u\frac{\partial C}{\partial x}+v\frac{\partial C}{\partial y}=D_{B}\frac{\partial^{2}C}{\partial y^{2}},$$

subject to:

(5) $$\begin{array}{l} t<0:\;\;u=0,\;\;T=T_{\infty },\;C=C_{\infty }\;\mathrm{for}\;\mathrm{any}\;\left(x,y\right),\\ t\geq 0:\;\;u=\lambda u_{w}\left(x,t\right),\;\;v=v_{w}\left(t\right),\;\;\;T=\;T_{w},\;C=C_{w}\;\;at\;\;y=0,\\ \;\;\;\;\;\;\;\;\;\;\;\;\;\;u\to 0,\;\;\;T\to T_{\infty },\;\;\;C=C_{\infty }\;\;\mathrm{as}\;\;y\to \infty . \end{array}$$

where (u,v), represent the velocity components, T the temperature, C the concentration, $Q_{0}$ the heat sink or source coefficient, and $K_{0}$ the essential permeability of the porous media. Besides, λ signifies the stretchable/shrinkable parameter, with λ=0 indicates the stationary sheet, while λ<0 and λ>0 indicate the shrinking and stretching sheet, respectively.

Table 1 gives the thermophysical properties of the water, Mn-ZnFe_2_O_4_, and CoFe_2_O_4_ nanomaterials [36,37]. Meanwhile, the correlations of the hybrid nanofluid are given in Equation (6) as follow [38,39,40]:

(6) $$\left\{ \begin{array}{l} \frac{k_{hnf}}{k_{f}}=\frac{\frac{\varphi_{1}k_{n1}+\varphi_{2}k_{n2}}{\varphi_{hnf}}+2k_{f}+2\left(\varphi_{1}k_{n1}+\varphi_{2}k_{n2}\right)-2\varphi_{hnf}k_{f}}{\frac{\varphi_{1}k_{n1}+\varphi_{2}k_{n2}}{\varphi_{hnf}}+2k_{f}-\left(\varphi_{1}k_{n1}+\varphi_{2}k_{n2}\right)+\varphi_{hnf}k_{f}},\\ {\left(\rho C_{p}\right)}_{hnf}=\left(1-\varphi_{hnf}\right){\left(\rho C_{p}\right)}_{f}+\varphi_{1}{\left(\rho C_{p}\right)}_{n1}+\varphi_{2}{\left(\rho C_{p}\right)}_{n2},\\ \rho_{hnf}=\left(1-\varphi_{hnf}\right)\rho_{f}+\varphi_{1}\rho_{n1}+\varphi_{2}\rho_{n2},\;\;\;\;\mu_{hnf}=\frac{\mu_{f}}{{\left(1-\varphi_{hnf}\right)}^{2.5}}, \end{array}\right.$$

where $\rho C_{p}$ is the heat capacity, k signifies the thermal conductivity, ρ represents the fluid density, and *μ* is the dynamic viscosity. Note that the symbols $\varphi_{1}$ and $\varphi_{2}$ denote the volume fraction of nanoparticles CoFe_2_O_4,_ and Mn-ZnFe_2_O_4,_ respectively, where $\varphi_{hnf}=\varphi_{1}+\varphi_{2}$. We also note that in Equation (6), the effective thermal conductivity does not consider the interfacial thermal resistance between the nanoparticles and the host fluid (see Ref. [41]).

The following similarity variables are employed in order to obtain the similarity solutions:

(7) $$\psi =\sqrt{\frac{a\nu_{f}}{1-\alpha t}}\;x\;f\left(\eta \right),\;\;\theta \left(\eta \right)=\;\frac{T-T_{\infty }}{T_{w}-T_{\infty }},\;\;\eta =y\sqrt{\frac{a}{\nu_{f}\left(1-\alpha t\right)}},\;\;\;\chi \left(\eta \right)=\frac{C-C_{\infty }}{C_{w}-C_{\infty }},$$

where a is a positive constant and ψ is the stream function, which is defined as

(8) $$u=\frac{\partial \psi }{\partial y}=\frac{ax}{1-\alpha t}f^{\prime }\left(\eta \right),\;v=\;-\frac{\partial \psi }{\partial x}=-\sqrt{\frac{a\nu_{f}}{1-\alpha t}}f\left(\eta \right),$$

which satisfies Equation (1). By setting η=0, the mass flux velocity $v_{w}(t)$ becomes:

(9) $$v_{w}\left(t\right)=-\sqrt{\frac{a\nu_{f}}{1-\alpha t}}S,$$

with the fluid kinematic viscosity $\nu_{f}={\left(\mu /\rho \right)}_{f}$. The mass flux parameter is denoted by f(0)=S, while S=0 signifies the impermeable surface. Moreover, S<0 and S>0 are for blowing and suction, respectively.

Substitute (7) and (8) into Equations (2)–(4), and the similarity ODEs are acquired as follows:

(10) $$\frac{\mu_{hnf}}{\mu_{f}}\frac{\rho_{f}}{\rho_{hnf}}f^{\prime \prime \prime }-{f^{\prime }}^{2}+{\mathrm{ff}}^{\prime \prime }-\frac{\beta }{2}\;\left(2f^{\prime }+\eta f^{\prime \prime }\right)-K\frac{\mu_{hnf}}{\mu_{f}}\frac{\rho_{f}}{\rho_{hnf}}f^{\prime }=0,$$

(11) $$\frac{k_{hnf}}{k_{f}}\frac{{\left(\rho C_{p}\right)}_{f}}{{\left(\rho C_{p}\right)}_{hnf}}\theta^{\prime \prime }+\frac{\mathrm{Pr}}{2}\left(2f-\eta \beta \right)\theta^{\prime }+\mathrm{Pr}\frac{{\left(\rho C_{p}\right)}_{f}}{{\left(\rho C_{p}\right)}_{hnf}}Q\theta =0,$$

(12) $$\frac{1}{Sc}\;\chi^{\prime \prime }+\;\frac{\chi^{\prime }}{2}\left(2f-\eta \beta \right)=0,$$

subject to BCs:

(13) $$\begin{array}{l} \;f\left(0\right)=S,\;\;f\left(0\right)=\lambda ,\;\theta \left(0\right)=1,\;\;\;\chi \left(0\right)=1,\\ f^{\prime }\left(\eta \right)\to 0,\;\;\;\theta \left(\eta \right)\to 0,\;\;\chi \left(\eta \right)\to 0\;\;as\;\;\;\eta \to \infty , \end{array}$$

where K is the porous medium parameter, Sc is the Schmidt number, Pr is the Prandtl number, Q is the heat sink or source parameter, and β is the unsteady parameter, defined as:

(14) $$Sc=\frac{\nu_{f}}{D_{B}},\;\;\;K=\frac{\nu_{f}}{aK_{0}},\;\;\;\mathrm{Pr}=\frac{{\left(\mu \;C_{p}\right)}_{f}}{k_{f}},\;\;Q=\frac{Q_{0}}{a{\left(\rho C_{p}\right)}_{f}},\;\;\;\beta =\frac{\alpha }{a}.$$

The parameter β shows the expansion/contraction strength. For a positive value of β>0, the stretching/shrinking surface becomes smaller with time, e.g., contracting, while for a negative value of β, the stretching/shrinking surface becomes larger with time, e.g., expanding (see Fang et al. [42]).

The physical quantities of practical interests are:

(15) $$C_{f}=\frac{\mu_{hnf}}{\rho_{f}u_{w}^{2}(x)}{\left(\frac{\partial u}{\partial y}\right)|}_{y=0},\;Nu_{x}=-\frac{x\;k_{hnf}}{(T_{\infty \;}-T_{w})k_{f}}\;{\left(\;\frac{\partial T}{\partial y}\right)|}_{y=0},Sh_{x}=-\frac{x\;}{\left(C_{\infty \;}-C_{w}\right)}{\left(\frac{\partial C}{\partial y}\right)|}_{y=0},$$

with $C_{f}$ representing the shear stress coefficient, $Nu_{x}$ the local Nusselt number, and $Sh_{x}$ the local Sherwood number. Using (7) and (15), one obtains:

(16) RexCf=μhnfμff″(0),    NuxRex=−khnfkfθ′(0),    ShxRex=−χ′(0),

where Rex=uw(x)x/νf signifies the local Reynolds number.

## 3. Time Stability Analysis

The time stability of the dual solutions is evaluated [43,44] by considering the following variables:

(17) $$\begin{array}{l} \psi =\sqrt{\frac{a\nu_{f}}{1-\alpha t}}\;x\;f\left(\eta ,\Gamma \right),\theta \left(\eta ,\Gamma \right)=\frac{T-T_{\infty }}{T_{w}-T_{\infty }},\Gamma =at,\\ \chi \left(\eta ,\Gamma \right)=\frac{C-C_{\infty }}{C_{w}-C_{\infty }},\;\eta =\sqrt{\frac{a/\nu_{f}}{\left(1-\alpha t\right)}}y, \end{array}$$

where

(18) $$u=\frac{ax}{1-\alpha t}\frac{\partial f}{\partial \eta }\left(\eta ,\Gamma \right),\;\;\;\;v=-\sqrt{\frac{a\nu_{f}}{1-\alpha t}}f\left(\eta ,\Gamma \right),$$

and Γ is dimensionless time.

Then, by using Equations (17) and (18), one obtains:

(19) $$\begin{array}{l} \frac{\mu_{hnf}}{\mu_{f}}\frac{\rho_{f}}{\rho_{hnf}}\frac{\partial^{3}f}{\partial \eta^{3}}+f\frac{\partial^{2}f}{\partial \eta^{2}}-{\left(\frac{\partial f}{\partial \eta }\right)}^{2}-\beta \;\left(\frac{\partial f}{\partial \eta }+\frac{\eta }{2}\;\frac{\partial^{2}f}{\partial \eta^{2}}\right)\\ \;\;\;\;\;\;\;\;\;\;\;\;\;\;\;\;\;\;\;\;\;\;\;\;\;\;\;\;\;\;\;\;\;\;\;\;\;\;\;\;\;\;\;\;\;\;\;\;-K\frac{\mu_{hnf}}{\mu_{f}}\frac{\rho_{f}}{\rho_{hnf}}\frac{\partial f}{\partial \eta }-(1+\beta \Gamma )\frac{\partial^{2}f}{\partial \eta \partial \Gamma }=0, \end{array}$$

(20) $$\frac{1}{\mathrm{Pr}}\frac{{\left(\rho C_{p}\right)}_{f}}{{\left(\rho C_{p}\right)}_{hnf}}\frac{k_{hnf}}{k_{f}}\frac{\partial^{2}\theta }{\partial \eta^{2}}+\left(f-\beta \;\frac{\eta }{2}\right)\frac{\partial \theta }{\partial \eta }+\frac{{\left(\rho C_{p}\right)}_{f}}{{\left(\rho C_{p}\right)}_{hnf}}Q\;\theta -(1+\beta \Gamma )\frac{\partial \theta }{\partial \Gamma }=0,$$

(21) $$\frac{1}{Sc}\;\frac{\partial^{2}\chi }{\partial \eta^{2}}+\left(f-\beta \frac{\eta }{2}\right)\;\frac{\partial \chi }{\partial \eta }-(1+\beta \Gamma )\frac{\partial \chi }{\partial \Gamma }=0,$$

subject to BCs:

(22) $$\begin{array}{l} \frac{\partial f}{\partial \eta }(0,\Gamma )=\lambda ,\;\;\;f(0,\Gamma )=S,\;\;\;\theta (0,\Gamma )=1,\;\;\;\chi (0,\Gamma )=1;\\ \frac{\partial f}{\partial \eta }(\eta ,\Gamma )\to 0,\;\;\;\theta (\eta ,\Gamma )\to 0,\;\;\;\chi (\eta ,\Gamma )\to 0\;\;\;\mathrm{as}\;\;\;\eta \to \infty . \end{array}$$

Then, consider the perturbation function [43]:

(23) $$\begin{array}{l} f(\eta ,\Gamma )=f_{0}(\eta )+e^{-\gamma \Gamma }F(\eta ,\Gamma ),\;\;\;\;\theta (\eta ,\Gamma )=\theta_{0}(\eta )+e^{-\gamma \Gamma }H(\eta ,\Gamma ),\\ \chi (\eta ,\Gamma )=\chi_{0}(\eta )+e^{-\gamma \Gamma }G(\eta ,\Gamma ), \end{array}$$

where F(η,Γ), H(η,Γ), and G(η,Γ) are arbitrary functions and are relatively small compared to $f_{0}(\eta )$, $\theta_{0}(\eta )$, and $\chi_{0}(\eta )$, and γ denotes the eigenvalue. By setting Γ=0, then $F(\eta ,\Gamma )=F_{0}(\eta )$, $H(\eta ,\Gamma )=H_{0}(\eta )$, and $G(\eta ,\Gamma )=G_{0}(\eta )$. Moreover, after linearization, the eigenvalue problems become

(24) $$\frac{\mu_{hnf}}{\mu_{f}}\frac{\rho_{f}}{\rho_{hnf}}F_{0}^{\prime \prime \prime }+\left(f_{0}F_{0}^{\prime \prime }+f_{0}^{\prime \prime }\;F_{0}\right)-2f_{0}^{\prime }\;F_{0}^{\prime }-\beta \left(F_{0}^{\prime }+\frac{\eta }{2}F_{0}^{\prime \prime }\right)-K\frac{\mu_{hnf}}{\mu_{f}}\frac{\rho_{f}}{\rho_{hnf}}F_{0}^{\prime }+\gamma F_{0}^{\prime }=0,$$

(25) $$\frac{1}{\mathrm{Pr}}\frac{k_{hnf}}{k_{f}}\frac{{\left(\rho C_{p}\right)}_{f}}{{\left(\rho C_{p}\right)}_{hnf}}H_{0}^{\prime \prime }+\left(f_{0}H_{0}^{\prime }+\theta_{0}^{\prime }F_{0}\right)-\beta \;\frac{\eta }{2}H_{0}^{\prime }+\frac{{\left(\rho C_{p}\right)}_{f}}{{\left(\rho C_{p}\right)}_{hnf}}QH_{0}+\gamma H_{0}=0,$$

(26) $$\frac{1}{Sc}\;G_{0}^{\prime \prime }+\left(f_{0}G_{0}^{\prime }+\chi_{0}^{\prime }F_{0}\right)-\beta \;\frac{\eta }{2}G_{0}^{\prime }+\gamma G_{0}=0,$$

subject to:

(27) $$\begin{array}{l} F_{0}^{\prime }(0)=0,\;\;\;F_{0}(0)=0,\;\;\;H_{0}(0)=0,\;\;\;G_{0}(0)=0,\\ F_{0}^{\prime }(\eta )\to 0,\;\;\;H_{0}(\eta )\to 0,\;\;\;G_{0}(\eta )\to 0\;\;\;\mathrm{as}\;\;\;\eta \to \infty . \end{array}$$

Here, to obtain γ from Equations (24)–(26), $F_{0}^{\prime }(\eta )\to 0\;\;\;\mathrm{as}\;\;\;\eta \to \infty$ in Equation (27) is substituted by $F^{\prime \prime }(0)=1$, as suggested by Harris et al. [45].

## 4. Analysis of Results and Discussion

Equations (10)–(13) were numerically solved by utilizing the bvp4c package available in MATLAB software. The detailed description of this method is given in [46]. The solver bvp4c is based on a finite difference method that employs the three-stage Lobatto IIIa formula, with fourth-order accuracy. To achieve the accuracy of the numerical values, the selection of the initial guess and the boundary layer thickness $\eta_{\infty }$ must be precise. Suitable finite values of η→∞, namely $\eta =\eta_{\infty }$ for the first solution, is within 6–10, while that of the second solution is in the range 20–50. The velocity, temperature, and concentration profile generated by the guess value must satisfy the far field boundary conditions (13) asymptotically. Determining an initial guess for the first solution is not difficult because the solution will converge rapidly, even using poor guesses. However, it is rather difficult to determine a sufficiently good guess for the second solution of Equations (10)–(13). In this case, we use the technique called continuation (Shampine et al. [46]).

Comparison is done for the physical quantity $f^{\prime \prime }(0)$ of Kameswaran et al. [47] for different values of K when $\varphi_{hnf}=S=\beta =0$, and λ=1, as shown in Table 2. A favorable agreement is found for all parameters considered. Table 3 presents the values of the related quantities, i.e., the mass transfer rate $-\chi^{\prime }(0)$, the heat transport rate $-\theta^{\prime }(0)$, and the friction factor $f^{\prime \prime }(0)$ for several values of the involved constraints when λ=−1 (shrinking sheet), Pr=6.2, and Sc=1. The consequence of increasing $\varphi_{hnf}$ is to augment the quantities of $f^{\prime \prime }(0)$ and $-\chi^{\prime }(0)$, but declines the quantities of $-\theta^{\prime }(0)$. Meanwhile, all these quantities enhance with the increase in K and S. Greater values of Q lower the heat transfer rate $-\theta^{\prime }(0)$, but the values of $f^{\prime \prime }(0)$ and $-\chi^{\prime }(0)$ are not influenced due to the fact that this parameter appears only in the energy equation. The value of $f^{\prime \prime }(0)$ decreases when the deceleration flow is stronger; however, the values of $-\theta^{\prime }(0)$ and $-\chi^{\prime }(0)$ enlarge.

**Figure 2:**
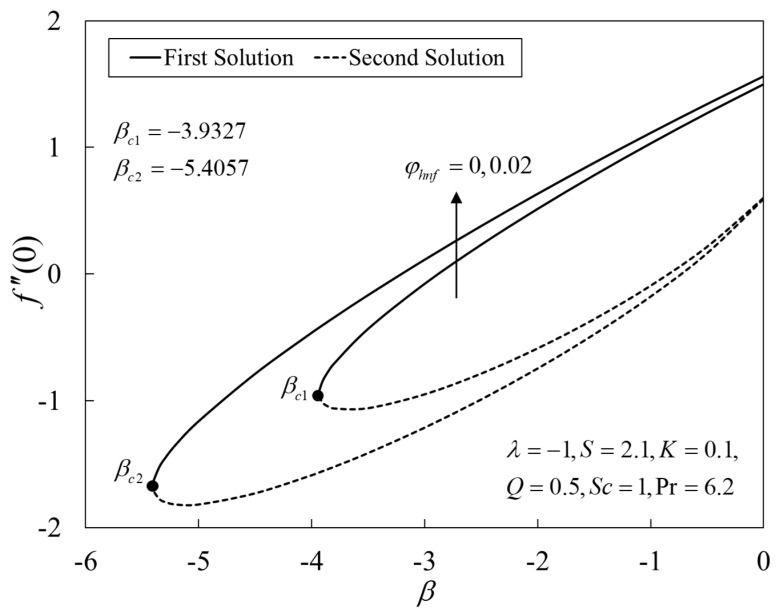
Plot of $f^{\prime \prime }(0)$ against *β*.

**Figure 3:**
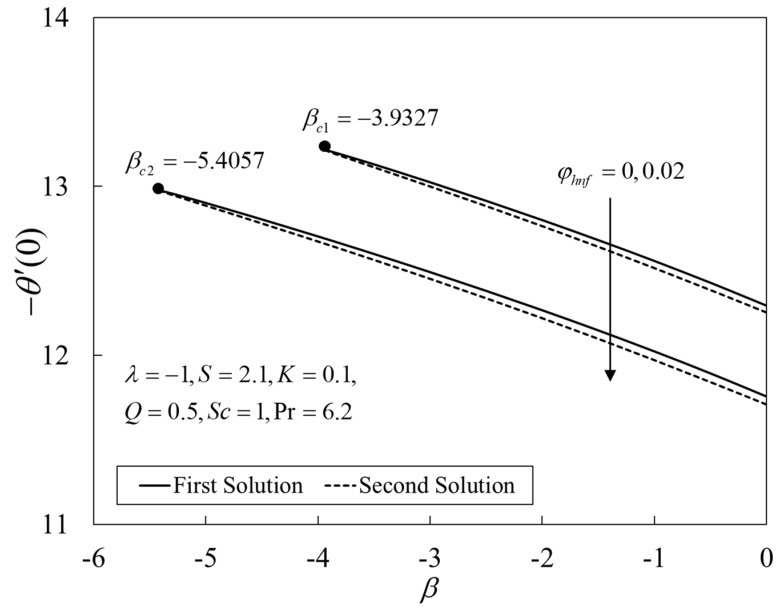
Plot of $-\theta^{\prime }(0)$ against β.

**Figure 4:**
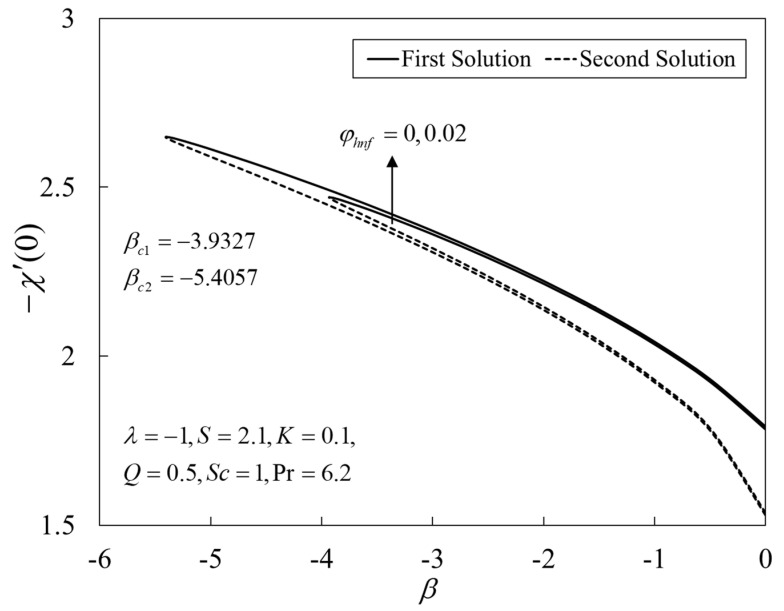
Plot of $-\chi^{\prime }(0)$ against β.

**Figure 5:**
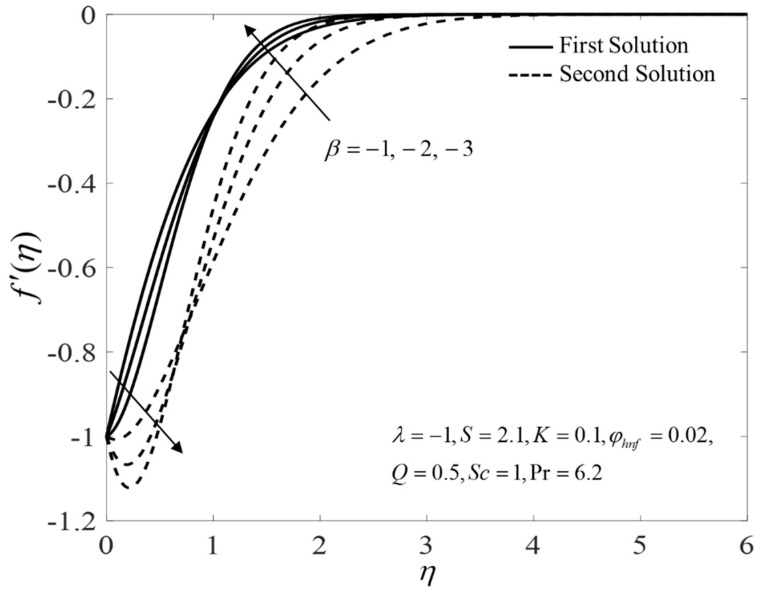
Plot of $f^{\prime }(\eta )$ for several values of β.

**Figure 6:**
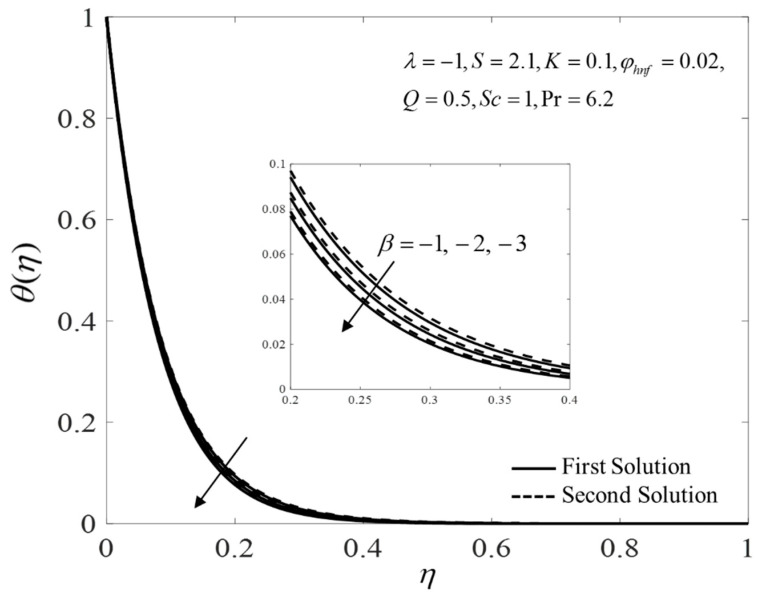
Plot of θ(η) for several values of β.

**Figure 7:**
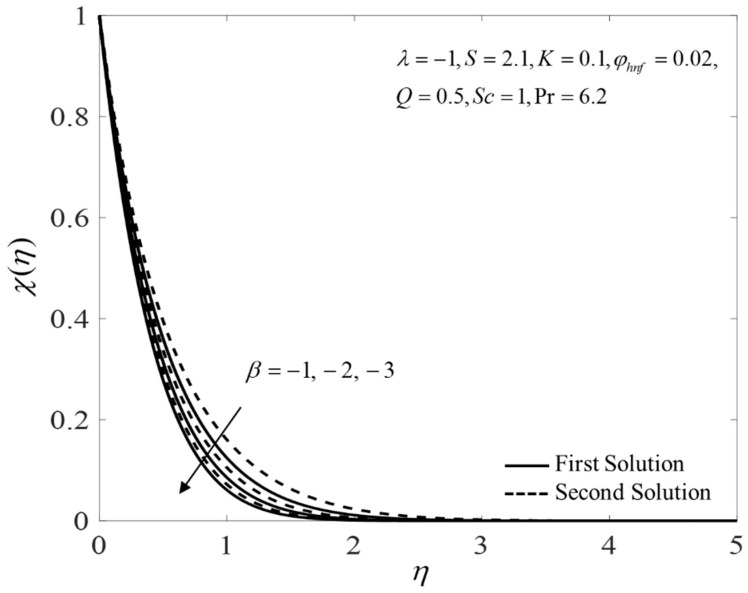
Plot of χ(η) for several values of β.

**Figure 8:**
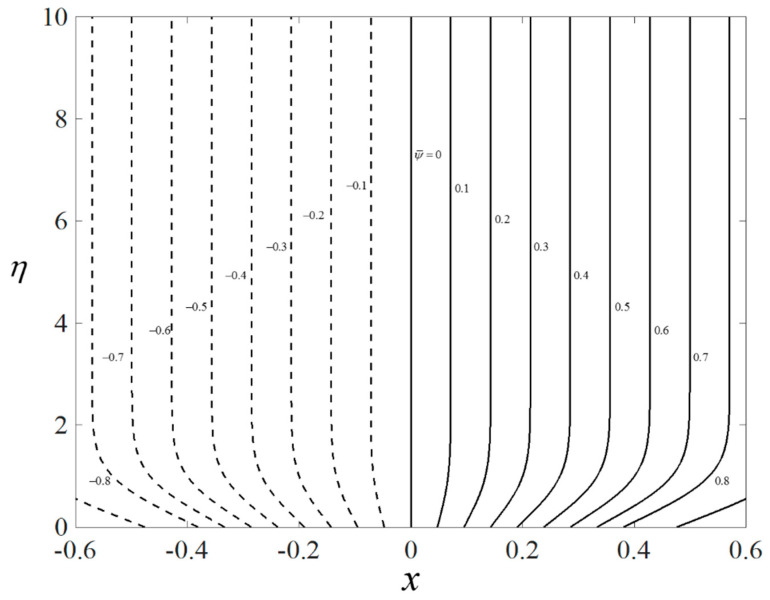
Plot of streamlines.

Figure 2, Figure 3 and Figure 4 present the outcomes of $f^{\prime \prime }(0)$, $-\theta^{\prime }(0)$, and $-\chi^{\prime }(0)$ for different choices of β for the viscous pure fluid $\left(\varphi_{hnf}=0\right)$ and the CoFe_2_O_4_-Mn-ZnFe_2_O_4_/water hybrid nanofluid $\left(\varphi_{hnf}=0.02\right)$. Note that the magnitude of $f^{\prime \prime }(0)$ and $-\chi^{\prime }(0)$ are higher, whereas $-\theta^{\prime }(0)$ is lower for a hybrid nanofluid. Physically, the fluid velocity reduces by adding nanoparticles into the fluid, which produces a higher velocity gradient. Moreover, this pertains to the concentration profile that leads to the increase in the mass transfer. The energy is dissipating in the form of heat, thus raising the fluid temperature. Due to this matter, the gradient of temperature decreases and thus lowers the heat transmission rate. The bifurcation, or critical values, climb with superior values of $\varphi_{hnf}$ with $\beta_{c1}=-3.9327$ $\left(\varphi_{hnf}=0\right)$ and $\beta_{c2}=-5.4057$ $\left(\varphi_{hnf}=0.02\right)$ that contribute to delay the separations of the boundary layer. In contrast, the rates of $-\theta^{\prime }(0)$ and $-\chi^{\prime }(0)$ lessen for the steady-state flow (β=0) and become higher when stronger deceleration flow is considered (β<0). However, the opposite behavior is seen for $f^{\prime \prime }(0)$. From these figures, it can be concluded that the deceleration flow is one of the factors in managing the mass and heat transfer of a hybrid nanofluid. Additionally, Figure 5, Figure 6 and Figure 7 are provided to show the effect of the unsteadiness parameter on the velocity, temperature, and concentration profiles. Meanwhile, Figure 8 represents the streamlines for the first solution.

**Figure 9:**
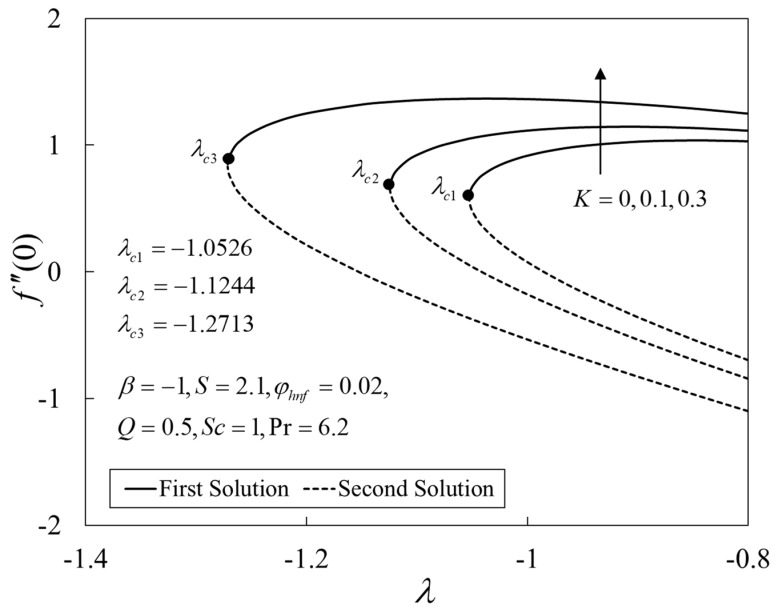
Plot of $f^{\prime \prime }(0)$ against *λ*.

**Figure 10:**
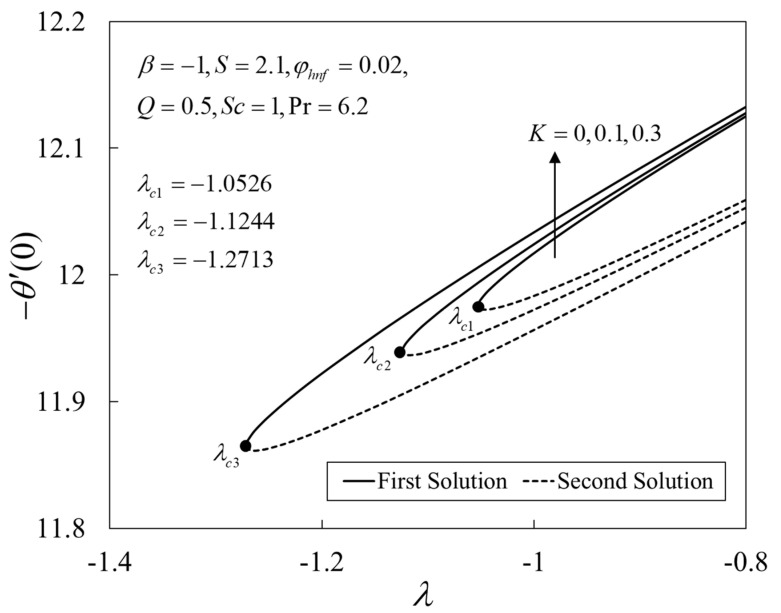
Plot of $-\theta^{\prime }(0)$ against λ.

**Figure 11:**
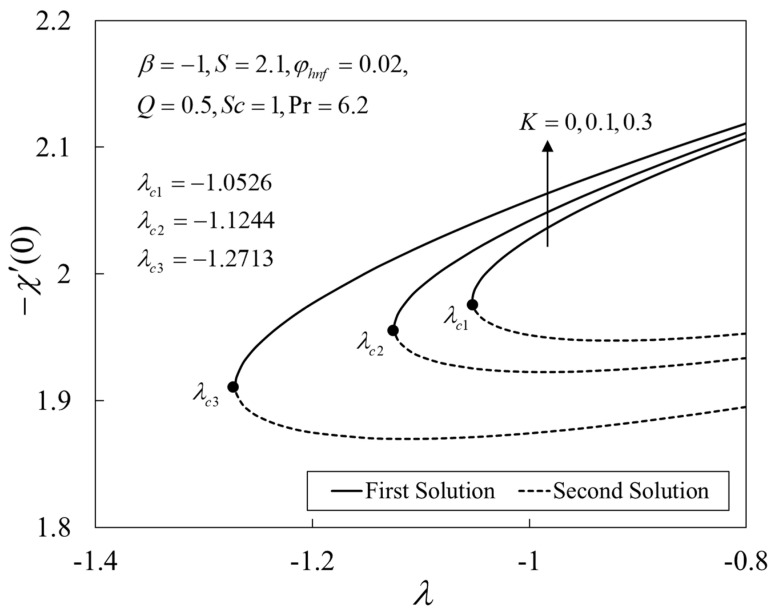
Plot of $-\chi^{\prime }(0)$ against λ.

Further, Figure 9, Figure 10 and Figure 11 show the deviations of $f^{\prime \prime }(0)$, $-\theta^{\prime }(0)$, and $-\chi^{\prime }(0)$ versus λ for several choices of K. The upshot of K is to boost the values of the physical amounts, such as the gradients. From the physical explanation, the porous medium amplifies the flow resistance, thus reducing the thickness of the momentum boundary layer by increasing its velocity on the shrinking sheet. Similar behaviors are seen for the temperature and the concentration profiles. This leads to the rise in the heat and mass transfers. Besides, the solutions terminate later for larger K, where the critical values are given by $\lambda_{c1}=-1.0526$ (K=0), $\lambda_{c2}=-1.1244$ (K=0.1), and $\lambda_{c3}=-1.2713$ (K=0.3).

**Figure 12:**
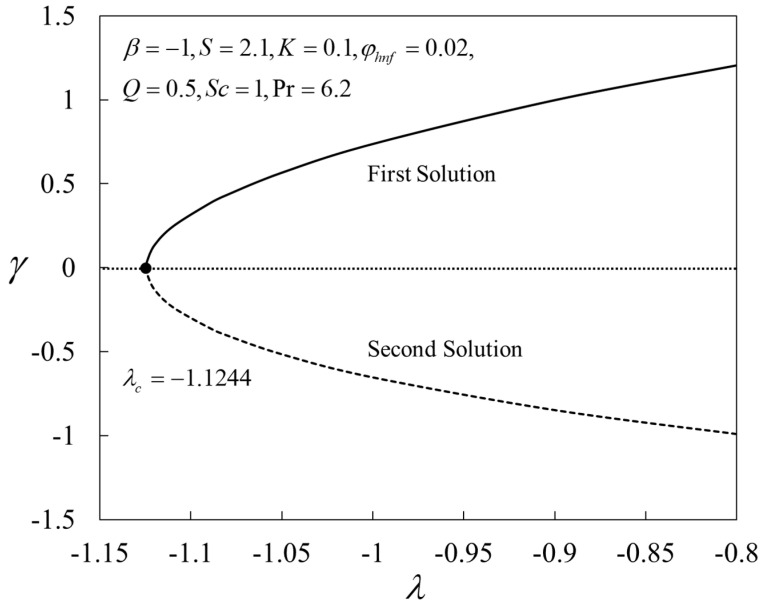
Plot of γ against λ.

The behaviors of the smallest eigenvalues γ against λ are displayed in Figure 12, where the first solutions represent positive eigenvalues, while the negative counterparts represent the second solution. Based on Equation (23), the first solution is stable, but the second solution is unstable as time evolves, Γ→∞.

This study offers valuable insight into fundamental transport phenomena, such as the transmission of momentum, heat, and mass. Thus, it provides valuable information on the gradients of essential factors to control the boundary layer flow pattern. The results would allow scientists and engineers to become familiar with the flow behavior of nanofluid and the way to predict the properties of this fluid for the possibility of using it in various engineering and industrial processes, such as blood flows, lubrication processes using grease and heavy oils, glass blowing, electronic chips, food stuff, slurries, etc.

## 5. Conclusions

The current study examined the time-varying flow over a shrinkable sheet with mixed ferrite nanoparticles, where the significant effects of porous media and the heat sinks/sources were taken into consideration. Numerical results for the similar equations were computed using MATLAB software. The key findings are summarized as:

- The mass transfer rate and the friction factor accelerate for the first branch solution, but decelerate for the second branch solution for high values of nanoparticle volume fraction $\varphi_{hnf}$, while the heat transfer rate abruptly diminished in both branch solutions.
- The heat and mass transfer rates, as well as the friction factor, enrich for the first solution for larger values of K, while the opposite is seen for the second solution.
- The magnitude of the critical values augments with larger values of $\varphi_{hnf}$ and K, which ultimately causes the separations in the boundary layer to diminish.
- The first branch solution shows an inertial decay of disturbance, which is thus physically reliable as time passes, whereas the second branch solution shows the opposite response.

## Figures and Tables

**Table 1:** The thermophysical properties.

Properties	*ρ*(kg/m^3^)	*C_p_*(J/kg K)	*k*(W/mk)	Pr
Water	997.1	4179	0.613	6.2
CoFe_2_O_4_	4907	700	3.7	
$\mathrm{Mn}-{\mathrm{ZnF}}_{2}O_{4}$	4900	800	5	

**Table 2:** Values of $-f^{\prime \prime }\left(0\right)$ for different values of K when $\varphi_{hnf}=S=\beta =0$, and λ=1.

K	Present Results	Kameswaran et al. [47]
0.5	1.22474487	1.22474487
1.0	1.41421356	1.41421356
1.5	1.58113883	1.58113883
2.0	1.73205081	1.73205081
5.0	2.44948974	2.44948974

**Table 3:** Values of the physical quantities for several choices of constraints when λ=−1 (shrinking sheet), Pr=6.2, and Sc=1.

$\varphi_{hnf}$	K	S	β	Q	$f^{\prime \prime }(0)$	$-\theta^{\prime }(0)$	$-\chi^{\prime }(0)$
0.00	0.1	2.1	−1.0	0.5	1.0326	12.5602	2.0355
0.01					1.0759	12.2887	2.0389
0.02					1.1129	12.0244	2.0418
0.02	0.0				0.9167	12.0170	2.0277
	0.2				1.2512	12.0295	2.0511
	0.3				1.3628	12.0335	2.0582
	0.1	2.15			1.2340	12.3364	2.0990
		2.2			1.3409	12.6469	2.1546
		2.3			1.5297	13.2649	2.2630
		2.1	−2.0		0.6321	12.2673	2.2218
			−3.0		0.1120	12.4926	2.3703
			−4.0		−0.4643	12.7034	2.4991
			−1.0	0.0	1.1129	12.2791	2.0418
				1.5	1.1129	11.4768	2.0418
				2.0	1.1129	11.1796	2.0418

## Data Availability

Not applicable.

## Nomenclature

a,α	constant
*C*	concentration
$C_{\infty }$	ambient concentration
$C_{w}$	surface concentration
$C_{f}$	skin friction coefficient
$C_{p}$	specific heat at constant pressure (J kg^−1^ K^−1^)
$D_{m}$	mass diffusivity coefficient
f	dimensionless stream function
F,G,H	arbitrary functions
$K_{0}$	permeability of the porous media
K	porous medium parameter
k	thermal conductivity of the fluid (W m^−1^ K^−1^)
$Nu_{x}$	local Nusselt number
Pr	Prandtl number
$Q_{0}$	heat sink or source coefficient
Q	heat sink or source parameter
Rex	local Reynolds number
S	mass flux velocity parameter
Sc	Schmidt number
$Sh_{x}$	local Sherwood number
t	time (s)
T	fluid temperature (K)
$T_{\infty }$	ambient temperature (K)
$T_{w}$	surface temperature (K)
u,v	velocity component in the *x-* and *y-* directions (m s^−1^)
$u_{w}$	velocity of the surface (m s^−1^)
$v_{w}$	velocity of the mass flux (m s^−1^)
x,y	Cartesian coordinates (m)
***Greek symbols***	
α	constant
β	unsteady parameter
Γ	dimensionless time variable
γ	eigenvalue
η	similarity variable
θ	dimensionless temperature
λ	stretching/shrinking parameter
μ	dynamic viscosity (kg m^−1^ s^−1^)
ν	kinematic viscosity of the fluid (m^2^ s^−1^)
ρ	density of the fluid (kg m^−3^)
$\varphi_{1}$	nanoparticle volume fractions for CoFe_2_O_4_ (cobalt ferrite)
$\varphi_{2}$	nanoparticle volume fractions for MnZnFe_2_O_4_ (manganese-zinc ferrite)
$\varphi_{hnf}$	hybrid nanoparticle volume fractions
χ	dimensionless concentration
ψ	stream function
***Subscripts***	
f	base fluid
hnf	hybrid nanofluid
n1	solid component for CoFe_2_O_4,_ (cobalt ferrite)
n2	solid component for MnZnFe_2_O_4_ (manganese-zinc ferrite)
***Superscript***	
′	differentiation with respect to η
